# Association Between Neutrophil-to-Lymphocyte Ratio and Vitamin D Deficiency in Patients With Sickle Cell Disease: A Cross-Sectional Study in Central Uganda

**DOI:** 10.7759/cureus.108469

**Published:** 2026-05-08

**Authors:** Enoch Muwanguzi, Jazira Tumusiime, Geofrey Salamu, Charles Nkubi Bagenda, Benson Okongo

**Affiliations:** 1 Department of Medical Laboratory Science, Mbarara University of Science and Technology, Mbarara, UGA; 2 Department of Medical Laboratory Science, Uganda Institute of Allied Health and Management Sciences, Kampala, UGA

**Keywords:** central uganda, deficiency, hematological ratios, insufficiency, vitamin d

## Abstract

Background: Vitamin D deficiency and chronic inflammation are prevalent in individuals with sickle cell disease (SCD), contributing to disease severity. Hematological ratios are simple inflammatory markers that can have various clinical outcomes. The main aim of our study was to assess the association between hematological ratios and vitamin D deficiency among patients with SCD in Central Uganda.

Methods: We conducted a cross-sectional study among patients with SCD attending Mengo Hospital, Central Uganda. Data were collected using a researcher-administered questionnaire supplemented with information from participants’ medical records. Anthropometric measurements were taken, and a blood sample was drawn for laboratory measurement of hematological parameters and serum vitamin D levels.

Results: Out of the 213 participants, 101 (47.4%) had serum 25-hydroxyvitamin D (25(OH)D) concentrations <20 ng/mL, indicating vitamin D deficiency, while 68 (31.9 %) had levels between 20-30 ng/mL, consistent with vitamin D insufficiency. Participants with vitamin D deficiency had a higher neutrophil-to-lymphocyte ratio (NLR) (0.27, IQR: 0.13-0.88) compared to those without vitamin D deficiency (0.22, IQR: 0.10-0.63), although the difference was not statistically significant (p=0.45). However, after adjusting for potential confounders, both the second (adjusted prevalence ratio (aPR)=1.83; 95% CI: 1.25-2.68; p=0.002) and third tertiles (aPR=1.74; 95% CI: 1.04-2.91; p=0.033) of the neutrophil-to-lymphocyte ratio were significantly associated with increased prevalence of vitamin D deficiency.

Conclusions: Vitamin D deficiency is prevalent among patients with SCD in Central Uganda. High NLR is potentially associated with vitamin D deficiency among patients with SCD.

## Introduction

Vitamin D deficiency, defined as serum 25-hydroxyvitamin D (25(OH)D) levels below 20 ng/mL [1], is a significant global health concern, and individuals living with sickle cell disease (SCD) are disproportionately affected [2]. Patients with SCD are particularly susceptible due to multiple interrelated factors, including increased melanin pigmentation, which reduces cutaneous vitamin D synthesis, chronic hemolysis, recurrent hospitalizations, poor nutritional intake, and reduced sunlight exposure resulting from limited outdoor activity [2-7]. This predisposition leads to considerably higher rates of vitamin D deficiency in patients with SCD compared to the general population. Global estimates indicate a prevalence of vitamin D deficiency among patients with SCD ranging from 56% to as high as 96%, far exceeding the rates observed in non-SCD populations [2,8]. Even in sun-rich sub-Saharan Africa, vitamin D insufficiency remains prevalent among SCD cohorts. For example, studies from Nigeria reported significantly lower mean vitamin D levels among SCD children compared to healthy controls [9,10], while among 4,509 children across Kenya, Uganda, Burkina Faso, Gambia, and South Africa, 0.6% had 25‑hydroxyvitamin D levels below 30 nmol/L and 7.8% were deficient (<50 nmol/L) [11]. In Uganda, studies in general populations have found vitamin D deficiency rates ranging from 9%-40% [12-14], yet data specifically among patients with SCD remain sparse, highlighting a critical knowledge gap in this region.

Vitamin D deficiency in SCD is not just a biochemical abnormality but has significant clinical consequences if left unaddressed. Vitamin D plays a crucial role in calcium metabolism and bone mineralization, and its deficiency aggravates the skeletal complications frequently seen in SCD, including osteomalacia, avascular necrosis, and chronic bone pain [2,15-17]. Beyond bone health, vitamin D has recognized immunomodulatory effects; deficiency promotes chronic inflammation, dysregulates T-cell responses, and impairs innate immune defense, predisposing patients with SCD to recurrent infections and more frequent vaso-occlusive crises [18-20]. In African settings, vitamin D deficiency has also been linked to increased risk of infections like tuberculosis and malaria [21,22], further underscoring its clinical relevance in SCD populations.

Emerging research indicates that hematological indices, particularly the neutrophil-to-lymphocyte ratio (NLR), may serve as accessible markers of systemic inflammation and immune dysregulation in chronic diseases [23-26]. In SCD, chronic hemolysis and inflammation typically manifest with elevated neutrophil counts, lymphopenia, and increased platelet counts, resulting in elevated hematological ratios like NLR and platelet-to-lymphocyte ratio (PLR) [27-30]. Simultaneously, vitamin D deficiency has been shown to correlate with elevated NLR in non-SCD populations, such as patients with type 2 diabetes and cardiovascular disease [31], suggesting that low levels of vitamin D may exacerbate systemic inflammation reflected in these hematological ratios. Mechanistically, vitamin D enhances anti-inflammatory pathways while suppressing pro-inflammatory cytokines and Th1/Th17 responses [32,33], providing biological plausibility for these associations. While such relationships have been explored in other diseases, limited evidence exists on the interplay between hematological ratios and vitamin D deficiency among patients with SCD, particularly in African settings. This constitutes a crucial gap in the literature, especially considering the high burden of both vitamin D deficiency and inflammatory complications among patients with SCD. To address this, we investigated associations between hematological ratios and vitamin D deficiency among patients with SCD attending a tertiary hospital in Central Uganda. This study provides novel insights into the potential utility of simple hematological markers in identifying vitamin D deficiency among patients with SCD in Central Uganda, a context where diagnostic resources are often limited.

## Materials and methods

This study was reported in accordance with the Strengthening the Reporting of Observational Studies in Epidemiology (STROBE) guidelines for observational studies [34].

Study design and setting

We conducted a hospital-based, descriptive and analytical cross-sectional study among patients with sickle cell disease (SCD) attending the sickle cell clinic at Mengo Hospital from June 2, 2025, to August 15, 2025. Mengo Hospital is a private, faith-based healthcare facility located on Namirembe Hill within Kampala City. Its catchment area includes Kampala Capital City Authority and surrounding divisions such as Lubaga, Nateete, Nakulabye, Makerere, and Busega, in addition to patients referred from other regions of the country. These areas are known to have a high prevalence of SCD, and Mengo Hospital is easily accessible to affected communities.

Study population, sampling, and eligibility criteria

The study population comprised children, adolescents, and adults with a confirmed diagnosis of SCD attending the Sickle Cell Clinic at Mengo Hospital during the study period. This included both previously diagnosed patients on routine follow-up and newly confirmed cases identified during the study period. A non-probability consecutive sampling approach was employed, whereby all eligible patients presenting to the clinic during routine visits within the study period were screened and recruited consecutively until the required sample size was attained. No randomization or selective recruitment procedures were applied.

Participants were eligible for inclusion if they had a documented confirmed diagnosis of SCD in their medical records, attended the clinic during the study period, and provided appropriate informed consent and/or assent according to age. Participants aged 18 years and above provided written informed consent directly. For participants below 18 years of age, written informed consent was obtained from a parent or legal caregiver, with additional age-appropriate assent obtained from children and adolescents aged 8-17 years. Participants were excluded if they had an acute illness or had received a blood transfusion within the preceding three months to avoid transient alterations in hematological indices attributable to donor cells, if they were receiving vitamin D supplementation at the time of recruitment due to its potential influence on serum vitamin D levels, or if consent and/or assent was not provided.

Sample Size Determination

We used the Kish-Leslie formula (1965) [35] to determine the minimum required sample size using the following assumptions: a 34.8% prevalence of vitamin D deficiency among sickle cell disease patients in Uganda [36], 5% precision, and a 95% confidence interval, with the Z-statistic of 1.96.

\[n = \frac{Z^{2}P(1 - P)}{d^{2}}\]

where n is required sample size, Z is Z-statistic for a level of confidence, P is expected prevalence, and d is the precision.

\[n = \frac{1.96^{2} \times 0.348(1 - 0.348)}{0.05^{2}} = 348\]

Taking 10% non-responsive rate into consideration, i.e., 0.1 × 348 = 34.8 participants. Therefore, the minimum sample size required = 348 + 34.8 = 383 participants. The Cochran's formula for finite population correction was applied to adjust the sample size.

\[ n = 1 + \frac{n_0}{\frac{n_0 - 1}{N}} \]

Where n = the reduced sample size, n_{0} = the estimated sample size (383), and N = estimated population with the SCD disease at Mengo Hospital (480).

\[n = 1 + \frac{383}{\frac{383 - 1}{480}}\]

n = 213. The study therefore recruited 213 study participants.

Ethical considerations

This study received ethical approval from the Research Ethics Committee [6] of Mbarara University of Science and Technology, Mbarara, Uganda, with approval number (MUST-2025-301) on May 27, 2025. Administrative clearance was also obtained from the Office of the Director, Mengo Hospital before conducting the study.

Consent to participate

Informed written consent was obtained prior to enrolment in the study. For participants aged 18 years and above, written informed consent was obtained directly from the participant. For participants between 8 to 17 years, written informed consent was obtained from a parent or legal caregiver and age-appropriate assent was also obtained from the participant. For participants below eight years, written informed consent was obtained from a parent or legal caregiver only.

The consent and assent forms were translated into the local language (Luganda) to ensure comprehension. Formally educated participants and caregivers provided consent or assent by signing the written forms. For participants or caregivers with no formal education, the written informed consent/assent form was read aloud in Luganda, followed by administration of the comprehension screening tool approved by the MUST Research Ethics Committee. Only those who demonstrated adequate understanding were allowed to provide consent or assent by placing a thumbprint on the informed consent/assent form.

The study protocol conformed to the ethical guidelines of the 1975 Declaration of Helsinki (1964). Confidentiality of the study participants was observed by giving each participant a study code that was not traceable to them, personal identifiers. Participants and caregivers were informed that participation was free and voluntary and that they had the right to withdraw from the study at any time without any effect on their care.

Study variables and data collection tools

Study Variables

Dependent variable: Serum vitamin D deficiency was the primary outcome variable. Blood samples were collected from each consented study participants in red top Vacutainers for the measurement of serum vitamin D levels. Samples were collected under routine, non-hemolyzed venous conditions, and visually inspected prior to processing. Samples showing gross hemolysis or lipemia were not processed. The collected samples were spun in a centrifuge at a speed of 3,200 rpm for five minutes to obtain serum. The serum samples were stored in a freezer at a temperature of -200°C, and tests were done within 24 hours of sample collection. The measurement was conducted using a well-calibrated and quality-controlled Cobas e-411 (Roche Diagnostics GmbH (Mannheim, Germany)) automated machine. Serum vitamin D levels were categorized as vitamin D deficiency (<20 ng/mL), vitamin D insufficiency (20-30 ng/mL), and normal serum vitamin D levels(>30 ng/mL) [1].

Independent variables: Hematological ratios were the major independent variables of this study. Blood samples were in purple top vacutainers for the measurement of full blood count using a well-calibrated and quality-controlled Mindray BC-6200 (Mindray BioMedical Electronics Co., Ltd., Shenzhen, China) automated hematology analyzer. The hematological parameters measured included white blood cell (WBC) count, neutrophils, monocytes, eosinophils, basophils, lymphocytes, red blood cell (RBC) count, hemoglobin concentration (g/dL), mean cell volume (MCV, fL), mean cell hemoglobin (MCH, pg), mean cell hemoglobin concentration (MCHC, g/dL), red cell distribution width coefficient of variation (RDW-CV, %), plateletcrit (PCT, %), platelet distribution width (PDW), platelet count, and hematocrit (HCT, %).

From the absolute counts of the WBC subtypes, platelet and hemoglobin concentration, the following hematological ratios were calculated: neutrophil-to-lymphocyte ratio (NLR), monocyte-to-lymphocyte ratio (MLR), eosinophil-to-lymphocyte ratio (ELR), basophil-to-lymphocyte ratio (BLR), platelet-to-lymphocyte ratio (PLR), and hemoglobin-to-RDW-CV ratio (HbRR). All derived ratios were categorized into tertiles for subsequent analysis (Appendix 1). The use of tertiles was chosen because there are no universally established or validated clinical cut-off values for these hematological ratios in patients with sickle cell disease, particularly within our study setting. In the absence of standardized thresholds, categorization into tertiles provided an objective, data-driven method to stratify participants into low, intermediate, and high groups. This approach facilitated clinically interpretable group comparisons, enabled assessment of potential dose-response trends across increasing ratio levels, and reduced the influence of extreme values and skewed distributions.

A structured questionnaire was administered to collect data on the social-demographic factors (age, gender, residence, family income per month), life style (vegetable, fruit, and meat consumption, dinner time, sleep duration, and physical activity), and clinical factors (systolic and diastolic blood pressure, hypertension, family history of chronic conditions; hypertension, kidney disease, cardiovascular disease, and dyslipidemia, history of cerebral malaria, SCD medication, duration on SCD medication, SCD crisis, use of lab monitoring tests. The questionnaire comprised standardized and previously validated tools for the measurement of the key abstract study variables. Physical activity was assessed using the International Physical Activity Questionnaires (IPAQ) [37] and categorized as <600, 600-3,000 and >3,000 metabolic equivalent of task (MET)-minutes/week [38]. Height and weight were measured using a digital balance with an attached height measurement and was used in the computation of body mass index (BMI). BMI was classified as underweight (BMI<18.5 kg/m^2^), normal weight (18.5-24.9 kg/m^2^), overweight (25-29.9 kg/m^2^), and obese (≥30 kg/m^2^) [39]. A digital sphygmomanometer was used to measure blood pressure. High blood pressure was defined as a systolic blood pressure of ≥140 mmHg and/or a diastolic blood pressure of ≥90 mmHg [40]. Low fruit and vegetable intake was defined as consuming less than five servings of fruit and vegetables per day [41]. Sleep duration was categorized as ≤7 hours and >7 hours.

Statistical Analysis

Data were entered into Microsoft Excel (Microsoft Corporation, Redmond, Washington, United States) 2019 and exported to STATA version 17 (StataCorp LLC, College Station, Texas, United States) for analysis. All participant data were fully de-identified prior to analysis. Personal identifiers were removed and replaced with unique study codes to ensure that individual participants could not be identified. The de-identified dataset was used for all statistical analyses. Continuous variables were assessed for normality using the Shapiro-Wilk test, and all were not normally distributed across the study participants (p-value<0.05). The continuous variables were summarized as medians (interquartile range, IQR) and compared across serum vitamin D level groups using the Kruskal Wallis test. Categorical variables were summarized as frequencies and percentages and compared using Chi-square or Fisher’s exact tests where appropriate. A p-value <0.05 was considered statistically significant. The proportion of participants with vitamin D deficiency was calculated as a percentage of the total participants. To assess the associations between independent variables and binary vitamin D deficiency, we performed a generalized linear model regression of the Poisson family with a log link (modified Poisson) using robust standard errors, as the prevalence of vitamin D deficiency exceeded 10%. Variables with p-values ≤0.2 at bivariate analysis and those with biological plausibility were included in the multivariate modified Poisson regression model. Hosmer-Lemeshow test was used to test the suitability of the final model in predicting the outcome variable (vitamin D deficiency). A p-value ≥0.05 indicated good goodness of fit of the final model. The final model was also tested for severe multicollinearity, and a mean variance inflation factor (VIF) of <5 was considered acceptable. Associations were considered statistically significant at p-value < 0.05.

## Results

Characteristics of the study participants

A total of 213 patients with SCD were enrolled, with a median age of 10 years (IQR: 5-13). A significant difference in age distribution was observed across vitamin D status groups (p<0.001), with the highest median age (12 years) among participants with vitamin D deficiency compared to 8.5 and 9 years in other groups. Gender distribution was relatively balanced, with males accounting for 50.7% (108/213) and females 49.3% (105/213), with no significant differences (p=0.731) across vitamin D level status. The majority resided in urban areas (74.2%, 158/213), and most households earned above 800,000 Ugandan shillings (UGX) monthly (56.3%, 120/213). About 76.5% (163/213) of the participants have a sleep duration of >7 hours. Notably, sleep duration showed a significant association (p=0.009); 32.7% of participants with vitamin D deficiency reported ≤7 hours of sleep, compared to 13.2% and 18.2% in other groups (Table 1).

**Table 1:** Socio-demographic and lifestyle characteristics of the study participants by serum vitamin D level status UGX: Ugandan shillings, MET: mitogen-activated protein kinase.

Variable Median (IQR), n (%)	Total N=213	Serum Vitamin D Level (ng/mL)			p-Value	Statistical Tests	Test Statistic Value
		<20 N=101	20-30 N=68	>30 N=44			
Age (years)	10 (5-13)	12 (6-14)	8.5 (5-11)	9 (3-13)	0.027	Kruskal-Wallis test	7.240
Age groups					<0.001	Fisher’s exact test	-
<12	131 (61.5%)	49 (48.5%)	54 (79.4%)	28 (63.6%)			
12-17	69 (32.4%)	41 (40.6%)	14 (20.6%)	14 (31.8%)			
≥18	13 (6.1%)	11 (10.9%)	0 (0.0%)	2 (4.5%)			
Gender					0.731	Chi-Square test	0.628
Male	108 (50.7%)	53 (52.5%)	35 (51.5%)	20 (45.5%)			
Female	105 (49.3%)	48 (47.5%)	33 (48.5%)	24 (54.5%)			
Residence					0.119	Chi-Square test	4.255
Rural	55 (25.8%)	32 (31.7%)	16 (23.5%)	7 (15.9%)			
Urban	158 (74.2%)	69 (68.3%)	52 (76.5%)	37 (84.1%)			
Family income per month (UGX)					0.798	Fisher’s exact test	-
<500,000	18 (8.5%)	7 (6.9%)	7 (10.3%)	4 (9.1%)			
500,000-800,000	75 (35.2%)	34 (33.7%)	23 (33.8%)	18 (40.9%)			
>800,000	120 (56.3%)	60 (59.4%)	38 (55.9%)	22 (50.0%)			
Vegetable and fruit intake per day					0.389	Chi-Square test	1.891
<5 servings	176 (82.6%)	87 (86.1%)	55 (80.9%)	34 (77.3%)			
≥5 servings	37 (17.4%)	14 (13.9%)	13 (19.1%)	10 (22.7%)			
Number of days of vegetable and fruit intake per week					0.237	Chi-Square test	2.883
≤4	155 (72.8%)	78 (77.2%)	49 (72.1%)	28 (63.6%)			
>4	58 (27.2%)	23 (22.8%)	19 (27.9%)	16 (36.4%)			
Meat consumption per month					0.823	Chi-Square test	1.523
None	27 (12.7%)	11 (10.9%)	11 (16.2%)	5 (11.4%)			
2-3 times	104 (48.8%)	51 (50.5%)	30 (44.1%)	23 (52.3%)			
≥4 times	82 (38.5%)	39 (38.6%)	27 (39.7%)	16 (36.4%)			
Dinner time					0.835	Chi-Square test	0.360
Before 8 pm	28 (32%)	13 (35%)	10 (29%)	5 (31%)			
After 8 pm	60 (68%)	24 (65%)	25 (71%)	11 (69%)			
Physical activity (MET mins/week)	456 (209-953)	495 (247-1,074)	493.5 (203.5-1,070.5)	349 (160.5-591.5)	0.059	Kruskal-Wallis test	5.677
Physical activity (MET mins/week)					0.101	Fisher’s exact test	-
<600	132 (62.0%)	58 (57.4%)	40 (58.8%)	34 (77.3%)			
600-3,000	64 (30.0%)	32 (31.7%)	22 (32.4%)	10 (22.7%)			
>3,000	17 ( 8.0%)	11 (10.9%)	6 (8.8%)	0 (0.0%)			
Body mass index (kg/m^2^)	17.28395 (15.11-21.89)	17.63 (15.63-20.83)	16.45 (14.62-23.76)	16.71 (14.41-21.82)	0.691	Kruskal-Wallis test	0.740
Body mass index (kg/m^2^)					0.533	Chi-Square test	-
<18.5	127 (59.6%)	59 (58.4%)	41 (60.3%)	27 (61.4%)			
18.5-24.9	50 (23.5%)	29 (28.7%)	12 (17.6%)	9 (20.5%)			
25-29.9	13 (6.1%)	5 (5.0%)	6 (8.8%)	2 (4.5%)			
≥30	23 (10.8%)	8 (7.9%)	9 (13.2%)	6 (13.6%)			
Sleep duration (hours)					0.009	Chi-Square test	9.412
≤7	50 (23.5%)	33 (32.7%)	9 (13.2%)	8 (18.2%)			
>7	163 (76.5%)	68 (67.3%)	59 (86.8%)	36 (81.8%)			

Clinical characteristics of the study participants by serum vitamin D level status

Majority of the participants had normal blood pressure, with 210 (98.6%) classified as normotensive and only three (1.4%) with high blood pressure, with no significant difference across vitamin D status groups (p=1.000). Significant differences were observed in the duration on sickle cell medication (p=0.041), where participants with vitamin D deficiency (<20 ng/mL) were more likely to have used medication for over 10 years (30.7%) compared to those with insufficient vitamin D levels (13.2%). Additionally, history of sickle cell crises significantly varied by vitamin D status (p=0.041), with 97.0% of those with vitamin D deficiency experiencing crises compared to 88.2% and 88.6% in other groups. Frequency of crises per year also differed significantly (p=0.012), with higher proportions of frequent crises among those with vitamin D deficiency (Table 2).

**Table 2:** Clinical characteristics of the study participants by serum vitamin D level status SCD: sickle cell disease, CVD: cardiovascular disease.

Variable Median (IQR), n (%)	Total N=213	Serum Vitamin D Level (ng/mL)			p-Value	Statistical Test	Test Statistic Value
		<20 N=101	20-30 N=68	>30 N=44			
Systolic blood pressure (mmHg)	111 (104-120)	113 (108-120)	110 (102-118.5)	114.5 (104-120)	0.234	Kruskal-Wallis test	2.904
Diastolic blood pressure (mmHg)	62 (60-66)	62 (60-66)	61.5 (59-66)	62 (60-66)	0.635	Kruskal-Wallis test	0.907
Blood pressure (mmHg)					1.000	Fisher’s exact test	-
Normal	210 (98.6%)	99 (98.0%)	67 (98.5%)	44 (100.0%)			
High	3 (1.4%)	2 (2.0%)	1 (1.5%)	0 (0.0%)			
Hypertension					0.757	Fisher’s exact test	-
No	196 (92.0%)	94 (93.1%)	61 (89.7%)	41 (93.2%)			
Yes	17 (8.0%)	7 (6.9%)	7 (10.3%)	3 (6.8%)			
Family history of hypertension					0.840	Chi-Square test	0.349
No	177 (83.1%)	85 (84.2%)	55 (80.9%)	37 (84.1%)			
Yes	36 (16.9%)	16 (15.8%)	13 (19.1%)	7 (15.9%)			
Family history of kidney disease					0.335	Fisher’s exact test	-
No	209 (98.1%)	100 (99.0%)	67 (98.5%)	42 (95.5%)			
Yes	4 (1.9%)	1 (1.0%)	1 (1.5%)	2 (4.5%)			
Family history of CVD					0.934	Fisher’s exact test	-
No	199 (93.4%)	95 (94.1%)	63 (92.6%)	41 (93.2%)			
Yes	14 (6.6%)	6 (5.9%)	5 (7.4%)	3 (6.8%)			
Family history of dyslipidemia					0.303	Fischer’s exact test	-
No	203 (95.3%)	98 (97.0%)	65 (95.6%)	40 (90.9%)			
Yes	10 (4.7%)	3 (3.0%)	3 (4.4%)	4 (9.1%)			
Cerebral malaria					0.406	Fisher’s exact test	-
No	197 (92.5%)	91 (90.1%)	65 (95.6%)	41 (93.2%)			
Yes	16 (7.5%)	10 (9.9%)	3 (4.4%)	3 (6.8%)			
Duration on SCD medication (years)	6 (3-11)	8 (4-12)	5 (3-8.5)	5 (2-12)	0.049	Kruskal-Wallis test	6.051
Duration on SCD medication (years)					0.041	Chi-Square test	9.939
<5	87 (40.8%)	35 (34.7%)	32 (47.1%)	20 (45.5%)			
5-10	72 (33.8%)	35 (34.7%)	27 (39.7%)	10 (22.7%)			
>10	54 (25.4%)	31 (30.7%)	9 (13.2%)	14 (31.8%)			
SCD medication					0.997	Fisher’s exact test	-
Folic acid only	85 (39.9%)	41 (40.6%)	27 (39.7%)	17 (38.6%)			
Hydroxyurea only	4 (1.9%)	2 (2.0%)	1 (1.5%)	1 (2.3%)			
Both folic acid and hydroxyurea	124 (58.2%)	58 (57.4%)	40 (58.8%)	26 (59.1%)			
SCD crisis					0.041	Fisher’s exact test	-
Yes	197 (92.5%)	98 (97.0%)	60 (88.2%)	39 (88.6%)			
No	16 (7.5%)	3 (3.0%)	8 (11.8%)	5 (11.4%)			
Number of times per year with SCD crisis					0.012	Fisher’s exact test	-
None	16 (7.5%)	3 (3.0%)	8 (11.8%)	5 (11.4%)			
Once	115 (54.0%)	51 (50.5%)	43 (63.2%)	21 (47.7%)			
More than once	82 (38.5%)	47 (46.5%)	17 (25.0%)	18 (40.9%)			
Lab monitoring tests					0.751	Fisher’s exact test	-
Yes	199 (93.4%)	95 (94.1%)	64 (94.1%)	40 (90.9%)			
No	14 (6.6%)	6 (5.9%)	4 (5.9%)	4 (9.1%)			

Among the 213 participants assessed, most hematological parameters did not differ significantly by vitamin D status. However, red cell distribution width-coefficient of variation (RDW-CV) and hemoglobin-to-RDW ratio (HbRR) showed statistically significant differences (p=0.038 and p=0.020, respectively). Participants with vitamin D deficiency (<20 ng/mL) had higher RDW-CV values (median: 25.2%) compared to those with sufficient levels (>30 ng/mL; median: 22.4%; p=0.038). Conversely, HbRR was significantly lower in the vitamin D-deficient group (median: 0.31) versus the sufficient group (median: 0.35; p=0.020). We did not observe a significant difference in the distribution of NLR across vitamin D status categories (p=0.451) as indicated in Table 3.

**Table 3:** Hematological parameters of the study participants by serum vitamin D level status All comparisons were performed using Kruskal-Wallis test. WBC: white blood cells, NEUT: neutrophils, MON: monocytes, EOS: eosinophils, BAS: basophils, LYMP: lymphocytes, NLR: neutrophil-to-lymphocyte ratio, MLR: monocyte-to-lymphocyte ratio, ELR: eosinophil-to-lymphocyte ratio, BLR: basophil-to-lymphocyte ratio, RBC: red blood cell count, Hb: hemoglobin concentration, MCV: mean corpuscular volume, MCH: mean corpuscular hemoglobin, MCHC: mean corpuscular hemoglobin concentration, RDW-CV: red cell distribution width-coefficient of variation, PCT: plateletcrit, PDW: platelet distribution width, PLT: platelet count, PLR: platelet-to-lymphocyte ratio, HCT: hematocrit, MPV: mean platelet volume, HbRR: hemoglobin red cell distribution ratio.

Variable: Median (IQR)	Total N=213	Serum Vitamin D Level (ng/mL)			p-Value	H-Statistical Value
		<20 N=101	20-30 N=68	>30 N=44		
WBC (×10^9^)/L	9.58 (6.55-13.69)	9.6 (6.56-14.5)	9.435 (6.19-13.645)	9.505 (6.875-12.34)	0.817	0.405
NEUT (×10^9^)/L	1.65 (0.59-4.66)	1.76 (0.73-4.88)	1.63 (0.53-5.64)	1.4 (0.52-3.79)	0.395	1.857
MON (×10^9^)/L	0.65 (0.37-1.01)	0.67 (0.36-1.00)	0.68 (0.39-1.33)	0.59 (0.32-0.98)	0.629	0.928
EOS (×10^9^)/L	0.26 (0.11-0.59)	0.24 (0.09-0.56)	0.24 (0.11-0.52)	0.40 (0.15-0.80)	0.074	5.201
BAS (×10^9^)/L	0.10 (0.06-0.15)	0.1 (0.06-0.16)	0.11 (0.07-0.15)	0.10 (0.07-0.16)	0.936	0.133
LYMP (×10^9^)/L	5.41 (3.81-7.73)	5.44 (3.97-8.3)	4.92 (3.48-7.28)	5.56 (3.79-8.14)	0.387	1.897
NLR	0.27 (0.11-0.81)	0.27 (0.13-0.88)	0.27 (0.10-0.99)	0.22 (0.10-0.63)	0.451	1.591
MLR	0.12 (0.07-0.20)	0.11 (0.07-0.19)	0.14 (0.07-0.21)	0.10 (0.06-0.19)	0.310	2.343
ELR	0.05 (0.02-0.10)	0.04 (0.02-0.09)	0.05 (0.02-0.10)	0.08 (0.03-0.12)	0.115	4.332
BLR	0.02 (0.01-0.03)	0.02 (0.01-0.03)	0.02 (0.02-0.03)	0.02 (0.01-0.02)	0.303	2.386
RBC (×10^12^)/L	2.71 (2.35-3.17)	2.63 (2.32-2.98)	2.77 (2.43-3.35)	2.86 (2.45-3.36)	0.063	5.525
Hb (g/dL)	7.9 (7.1-9.1)	7.8 (6.9-8.8)	8.1 (7.4-9.3)	7.9 (7.15-9.95)	0.110	4.423
MCV (fL)	88.5 (80.6-96.8)	90 (83.2-98.8)	86.9 (79.95-95.35)	87.65 (80.1-93.6)	0.258	2.708
MCH (pg)	29.5 (26.4-32.3)	29.9 (26.7-33.2)	28.9 (26.3-32.3)	29.5 (26.65-31)	0.566	1.139
MCHC (g/dL)	33.2 (32.1-34.1)	33 (31.8-34)	33.2 (32.3-34.2)	33.2 (32-34.05)	0.287	2.500
RDW-CV (%)	23.4 (20-28.2)	25.2 (20.7-30.6)	22.4 (19.65-26.2)	22.4 (20.1-27.2)	0.038	6.547
PCT (%)	0.36 (0.24-0.50)	0.37 (0.22-0.53)	0.39 (0.26-0.50)	0.33 (0.22-0.45)	0.486	1.444
PDW	15.6 (15.3-16.1)	15.7 (15.3-16.2)	15.6 (15.3-16)	15.6 (15.35-15.95)	0.461	1.549
PLT (×10^9^)/L	369 (254-515)	381 (259-537)	369 (263.5-508.5)	346 (222.5-461.5)	0.437	1.655
PLR	62.31 (41.34-111.92)	63.46 (39.21-117.27)	61.84 (45.48-119.37)	62.09 (37.17-97.86)	0.616	0.970
HCT (%)	24 (21.6-27.1)	23.8 (21.3-26.6)	24.35 (21.8-28.15)	23.9 (21.35-29.5)	0.273	2.594
MPV (fL)	9.8 (8.9-10.7)	9.8 (9-10.4)	9.95 (8.75-10.85)	9.95 (8.8-10.6)	0.884	0.246
HbRR	0.34 (0.25-0.43)	0.31 (0.23-0.40)	0.37 (0.28-0.46)	0.35 (0.27-0.48)	0.020	7.871

Association between neutrophil-to-lymphocyte ratio and vitamin D deficiency among patients with SCD

At bivariate analysis (Table 4), a higher NLR was significantly associated with vitamin D deficiency among patients with SCD. Participants in the second NLR tertile had a 52% higher prevalence of vitamin D deficiency compared to the first tertile (crude prevalence ratio (cPR)=1.52; 95% CI: 1.06-2.17; p=0.022), while the third tertile showed no significant association (p=0.312). However, after adjusting for potential confounders in multivariate analysis (Table 4), both the second (adjusted prevalence ratio (aPR)=1.83; 95% CI: 1.25-2.68; p=0.002) and third tertiles (aPR=1.74; 95% CI: 1.04-2.91; p=0.033) remained significantly associated with increased prevalence of vitamin D deficiency. Age was another independent factor, with adolescents (12-17 years) and adults (≥18 years) showing significantly higher prevalence of vitamin D deficiency compared to younger children (<12 years), with adjusted prevalence ratios of 1.94 (p=0.009) and 3.88 (p=0.001), respectively. Additionally, sleep duration (≤7 hours) and rural residence were significantly associated with a higher vitamin D deficiency at bivariate analysis (cPR=1.58, p=0.001 and cPR=1.33, p=0.049, respectively), but these associations lost statistical significance after adjustment in multivariate model (p=0.160 and p=0.573).

**Table 4:** Factors associated with vitamin D deficiency among patients with SCD Statistical model: Generalized linear model regression of the Poisson family with a log link (modified Poisson) using robust standard errors. Final multivariate model: Vitamin D deficiency + NLR + PLR + MLR + ELR + BLR + HbRR + Age + Gender + residence + BMI + Blood pressure + Sleep duration + Duration on SCD medication + SCD medication + SCD crisis + rbc. The rest of the variables with no multivariate data were not included in the final multivariate model. cPR: crude prevalence ratio, aPR: adjusted prevalence ratio, NLR: neutrophil-to-lymphocyte ratio, PLR: platelet-to-lymphocyte ratio, MLR: monocyte-to-lymphocyte ratio, ELR: eosinophil-to-lymphocyte ratio, BLR: basophil-to-lymphocyte ratio, HbRR: hemoglobin-to-RDW-CV ratio, SCD: sickle cell disease, UGX: Ugandan Shillings, MET: metabolic equivalent of task, CVD: cardiovascular disease.

Variable	Bivariate		Multivariate	
	cPR (95%CI)	p-Value	aPR (95%CI)	p-Value
NLR tertiles				
First	1.00		1.00	
Second	1.52 (1.06-2.17)	0.022	1.83 (1.25-2.68)	0.002
Third	1.22 (0.83-1.80)	0.312	1.74 (1.04-2.91)	0.033
PLR tertile				
First	1.00		1.00	
Second	0.94 (0.66-1.34)	0.737	0.89 (0.59-1.34)	0.574
Third	1.03 (0.73-1.45)	0.867	0.82 (0.52-1.30)	0.401
MLR tertiles				
First	1.00		1.00	
Second	1.00 (0.71-1.40)	1.000	0.82 (0.54-1.27)	0.381
Third	0.89 (0.62-1.26)	0.503	0.76 (0.43-1.34)	0.337
ELR tertiles				
First	1.00		1.00	
Second	1.06 (0.77-1.45)	0.738	1.19 (0.79-1.81)	0.401
Third	0.75 (0.51-1.09)	0.134	0.93 (0.55-1.58)	0.783
BLR tertiles				
First	1.00		1.00	
Second	0.79 (0.56-1.12)	0.184	0.79 (0.54-1.15)	0.213
Third	0.87 (0.62-1.21)	0.404	0.71 (0.49-1.05)	0.085
HbRR tertiles				
First	1.00		1.00	
Second	0.74 (0.54-1.02)	0.069	0.81 (0.56-1.18)	0.278
Third	0.60 (0.42-0.87)	0.006	0.78 (0.48-1.25)	0.297
Age (years)				
<12	1.00		1.00	
12-17	1.59 (1.18-2.14)	0.002	1.94 (1.18-3.20)	0.009
≥18	2.26 (1.64-3.12)	<0.001	3.88 (1.72-8.76)	0.001
Gender				
Male	1.00		1.00	
Female	0.93 (0.70-1.24)	0.625	0.84 (0.63-1.12)	0.241
Residence				
Rural	1.33 (1.00-1.77)	0.049	1.09 (0.81-1.46)	0.573
Urban	1.00		1.00	
Family income per month (UGX)				
<500,000	0.78 (0.42-1.43)	0.418	-	-
500,000-800,000	0.91 (0.67-1.23)	0.532	-	-
>800,000	1.00			
Vegetable and fruit intake				
<5 servings per day	1.31 (0.84-2.03)	0.234	-	-
≥5 servings per day	1.00			
Number of days of vegetable and fruit intake				
≤4 days	1.27 (0.89-1.81)	0.188	-	-
>4 days	1.00			
Meat consumption per month				
None	1.00			
2-3 times	1.20 (0.73-1.98)	0.464	-	-
≥4 times	1.17 (0.70-1.94)	0.552	-	-
Physical activity (MET mins/week)				
<600	0.88 (0.64-1.20)	0.418	-	-
600-3000	1.00			
>3,000	1.29 (0.84-1.99)	0.239	-	-
Body mass index (kg/m^2^)				
<18.5	0.80 (0.59-1.08)	0.149	0.90 (0.66-1.23)	0.529
18.5-24.9	1.00		1.00	
25-29.9	0.66 (0.32-1.37)	0.269	0.96 (0.47-1.99)	0.920
≥30	0.60 (0.33-1.10)	0.100	0.71 (0.40-1.28)	0.258
Blood pressure (mmHg)				
Normal	1.00		1.00	
High	1.41 (0.63-3.19)	0.405	0.69 (0.29-1.64)	0.404
Sleep duration				
≤7 hours	1.58 (1.21-2.07)	0.001	1.24 (0.92-1.68)	0.160
>7 hours	1.00		1.00	
Hypertension				
No	1.00			
Yes	0.86 (0.48-1.55)	0.611	-	-
Family history of hypertension				
No	1.00			
Yes	0.93 (0.62-1.38)	0.702	-	-
Family history of kidney disease				
No	1.00			
Yes	0.52 (0.09-2.88)	0.456	-	-
Family history of CVD				
No	1.00			
Yes	0.90 (0.48-1.67)	0.735	-	-
Family history of dyslipidemia				
No	1.00			
Yes	0.62 (0.24-1.62)	0.331	-	-
History of cerebral malaria				
No	1.00			
Yes	1.35 (0.90-2.04)	0.148	-	-
Duration on SCD medication (years)				
<5	1.00		1.00	
5-10	1.21 (0.85-1.71)	0.289	1.04 (0.70-1.55)	0.847
>10	1.43 (1.01-2.01)	0.043	0.68 (0.37-1.25)	0.216
SCD medication				
Folic acid only	1.00		1.00	
Hydroxyurea only	1.04 (0.38-2.84)	0.944	1.25 (0.48-3.25)	0.646
Both folic acid and hydroxyurea	0.97 (0.73-1.30)	0.835	0.74 (0.53-1.04)	0.084
SCD crisis				
Yes	2.65 (0.95-7.45)	0.064	2.04 (0.72-5.08)	0.181
No	1.00		1.00	
Lab monitoring tests				
Yes	1.00			
No	0.90 (0.48-1.67)	0.735	-	-
WBC (×10^9^)/L	1.01 (1.00-1.020	0.216	-	-
RBC (×10^12^)/L	0.77 (0.63-0.94)	0.009	0.88 (0.72-1.07)	0.194

## Discussion

Our finding that Ugandan patients with SCD with a higher neutrophil-to-lymphocyte ratio (NLR) are significantly more likely to have vitamin D deficiency carries important clinical implications. The NLR serves as a simple, accessible index of systemic inflammation and disease activity in SCD [42]. Even in equatorial Uganda, where sunlight is abundant, healthy children show 15% prevalence of vitamin D deficiency [43]; consequently, patients with SCD likely face an even higher risk of deficiency. Vitamin D deficiency is linked to worsened SCD outcomes, including more frequent pain crises [17]. In fact, a pediatric study showed that normalizing vitamin D levels significantly reduced pain-related emergency visits in SCD [44]. Thus, our results suggest that patients with a heightened inflammatory state (reflected by a higher NLR) are at higher risk of low vitamin D, with potential clinical consequences. These findings imply that routine screening of vitamin D status in patients with SCD with high NLR could help identify those needing supplementation to mitigate inflammation-driven complications and improve outcomes.

Biologically, this association is plausible because vitamin D exerts potent anti-inflammatory effects. Activated vitamin D interacts with immune and endothelial cells to downregulating inflammatory cascades. For example, vitamin D inhibits the nuclear factor kappa-light-chain-enhancer of activated B cells (NF-κB) and mitogen-activated protein kinase (MAPK) pathways, reducing production of tumor necrosis factor-α (TNF-α), interleukin 6 (IL-6), IL-1β and other pro-inflammatory cytokines while promoting IL-10 [45,46]. It also suppresses expression of endothelial adhesion molecules, thereby reducing leukocyte adhesion and neutrophil trafficking [45,47]. Additionally, vitamin D induces expression of antimicrobial peptides such as cathelicidin in neutrophils, enhancing pathogen defense without excessive inflammation [45,48]. Vitamin D also upregulates antioxidant enzymes such as glutathione and superoxide dismutase [49], which can curb neutrophil oxidative burst. In SCD, where neutrophil adhesion contributes to vaso-occlusion, insufficient vitamin D would remove these regulatory brakes, favoring a neutrophil predominance and elevating the NLR. Thus, vitamin D deficiency can exacerbate the chronic inflammatory milieu in SCD, making the observed NLR association biologically coherent.

Our findings contribute to a complex and sometimes contradictory body of literature. For example, Akbas et al. reported that NLR was significantly higher in vitamin D-deficient patients compared to non-deficient individuals [50], consistent with our direction. Likewise, a pediatric SCD cohort showed that vitamin D-deficient patients had elevated pro-inflammatory cytokine profiles, which normalized after three months of vitamin D supplementation [17]. In contrast, some studies report no link between vitamin D and NLR: Yilmaz et al. found no significant correlation in elderly subjects [51], and Dziedzic et al. saw no association in coronary artery disease patients [52]. Similarly, a study of SCD children by Oztas et al. found that baseline inflammatory markers did not differ by vitamin D status [53], although acute chest events were more frequent in the deficient group. These discrepancies may reflect differences in population, disease context, and methods: for instance, the study by Yilmaz et al. used a 30 ng/mL cutoff for deficiency [51], whereas our study used 20 ng/mL. Other factors like age, genetics, or comorbid infections could also influence results. Local factors such as endemic infections or malnutrition might elevate both NLR and vitamin D deficiency simultaneously, reinforcing this link in our population. Overall, our study extends prior work by demonstrating an NLR-vitamin D link in SCD, but variable findings across settings highlight the need for context-specific investigations.

Our study has some limitations that should be considered when interpreting the findings. First, the cross-sectional design provides only a snapshot of the prevalence of vitamin D deficiency and its association with elevated NLR, RDW-CV, and advancing age, thereby limiting the ability to establish causality or determine the temporal sequence of these relationships. In particular, although age was adjusted for in multivariable analyses, residual age-dependent confounding cannot be entirely excluded, especially given the higher burden of vitamin D deficiency observed among older participants. Second, some variables, including family history of hypertension, chronic kidney disease, and cardiovascular disease, were self-reported and may therefore be subject to recall bias or misclassification.

Although standardized laboratory procedures and internal quality control measures were implemented for serum 25-hydroxyvitamin D (25(OH)D) analysis, residual pre-analytical and analytical variability cannot be entirely excluded. Such variability may have potentially influenced the observed association between vitamin D status and RDW-CV. Furthermore, RDW-CV is a non-specific hematological parameter influenced by multiple factors such as iron status, inflammation, hemolysis, and bone marrow activity, which may act as residual confounders despite our exclusion criteria. An additional limitation of this study relates to the issue of multiple statistical testing. We examined several hematological ratios (NLR, PLR, MLR, ELR, BLR, and HbRR) in relation to vitamin D deficiency, which may potentially increase the risk of type I error due to multiple comparisons. Although ratios were selected a priori based on biological plausibility, no formal correction for multiple testing was applied. The categorization of hematological ratios into tertiles, although data-driven and used in the absence of established clinical cut-offs, may have reduced statistical power and obscured potential linear relationships. Furthermore, the use of a non-probability consecutive sampling approach may limit representativeness and introduce selection bias, thereby affecting generalizability of the findings.

Finally, as this was a single-center study, the findings may not be fully generalizable to other sickle cell populations with different demographic or clinical characteristics. The use of non-probability consecutive sampling is a potential source of selection bias, and the sampled participants may not fully represent the wider SCD population outside those attending routine clinic visits at this facility. The SCD genotype/subtype was not recorded, and the absence of this information may limit interpretation of the findings since variation in SCD subtype could influence hematological markers and vitamin D status. In addition, categorizing continuous variables into tertiles may have reduced statistical power and obscured or distorted true associations. These limitations underscore the need for larger, multi-center, and longitudinal studies to better clarify the relationship between vitamin D status and NLR and hematological parameters in sickle cell disease.

## Conclusions

Vitamin D deficiency is prevalent among patients with SCD in Central Uganda. High NLR is potentially associated with vitamin D deficiency among patients with SCD. While the association between vitamin D status and disease severity remains an area of ongoing investigation, our findings suggest that vitamin D deficiency represents a potentially modifiable factor in this population. In this context, vitamin D supplementation may serve as a simple and practical public health intervention that could contribute to improved clinical outcomes if a causal relationship is confirmed. We recommend future longitudinal and interventional studies to further explore the association between hematological ratios and vitamin D deficiency and to evaluate the clinical benefits of vitamin D supplementation in this unique population.

## Appendices

Appendix 1 

**Table 5:** Lower and upper limits of hematological ratios in each tertile NLR: neutrophil-to-lymphocyte ratio, PLR: platelet-to-lymphocyte ratio, MLR: monocyte-to-lymphocyte ratio, ELR: eosinophil-to-lymphocyte ratio, BLR: basophil-to-lymphocyte ratio, HbRR: hemoglobin-to-RDW-CV ratio.

Hematological Ratio	Lower Limit	Upper Limit
NLR		
First tertile	0.0189573	0.1387435
Second tertile	0.1424288	0.5866365
Third tertile	0.6165312	7.5040000
PLR		
First tertile	4.8736160	47.41379
Second tertile	48.347830	85.75419
Third tertile	86.911000	316.9742
MLR		
First tertile	0.0006386	0.0821256
Second tertile	0.0835165	0.1731959
Third tertile	0.1764706	2.1284400
ELR		
First tertile	0.0000000	0.0276923
Second tertile	0.0282258	0.0822060
Third tertile	0.0831325	0.3934066
BLR		
First tertile	0.0027933	0.0149051
Second tertile	0.0149254	0.0221811
Third tertile	0.0222222	0.324159
HbRR		
First tertile	0.0380208	0.2803030
Second tertile	0.2836879	0.3942308
Third tertile	0.3957447	0.8300654
